# Potential therapeutic strategies for non - muscle invasive bladder cancer based on association of intravesical immunotherapy with P-MAPA and systemic administration of cisplatin and doxorubicin

**DOI:** 10.1590/S1677-5538.IBJU.2015.0381

**Published:** 2016

**Authors:** Queila Cristina Dias, Iseu da Silva Nunes, Patrick Vianna Garcia, Wagner José Fávaro

**Affiliations:** 1 Laboratório de Urogenital Carcinogênese e Imunoterapia do Departamento de Biologia Estrutural e Funcional da Universidade de Campinas (UNICAMP), Campinas, SP, Brasil; 2 FarmaBrasilis R & D, Campinas, SP, Brasil

**Keywords:** Urinary Bladder Neoplasms, Immunotherapy, Cisplatin, Doxorubicin

## Abstract

The present study describes the histopathological and molecular effects of P-MAPA (Protein aggregate magnesium-ammonium phospholinoleate-palmitoleate anhydride) intravesical immunotherapy combined with systemic doxorubicin or cisplatin for treatment of non-muscle invasive bladder cancer (NMIBC) in an appropriate animal model. Our results showed an undifferentiated tumor, characterizing a tumor invading mucosa or submucosa of the bladder wall (pT1) and papillary carcinoma in situ (pTa) in the Cancer group. The histopathological changes were similar between the combined treatment with intravesical P-MAPA plus systemic Cisplatin and P-MAPA immunotherapy alone, showing decrease of urothelial neoplastic lesions progression and histopathological recovery in 80% of the animals. The animals treated systemically with cisplatin or doxorubicin singly, showed 100% of malignant lesions in the urinary bladder. Furthemore, the combined treatment with P-MAPA and Doxorubicin showed no decrease of urothelial neoplastic lesions progression and histopathological recovery. Furthermore, Akt, PI3K, NF-kB and VEGF protein levels were significantly lower in intravesical P-MAPA plus systemic cisplatin and in intravesical P-MAPA alone treatments than other groups. In contrast, PTEN protein levels were significantly higher in intravesical P-MAPA plus systemic cisplatin and in intravesical P-MAPA alone treatments. Thus, it could be concluded that combination of intravesical P-MAPA immunotherapy and systemic cisplatin in the NMIBC animal model was effective, well tolerated and showed no apparent signs of antagonism between the drugs. In addition, intravesical P-MAPA immunotherapy may be considered as a valuable option for treatment of BCG unresponsive patients that unmet the criteria for early cystectomy.

## INTRODUCTION

The primary treatment for high grade nonmuscle invasive bladder cancer (NMIBC) is based on surgery by Transurethral Resection of Bladder Tumor (TURBT), followed by intravesical immunotherapy with Bacillus Calmette-Guerin (BCG) to prevent recurrence and reduct the tumor progression (1). However, undesirable side events related with BCG therapy are observed up to 90% of patients and range from cystitis and irritative voiding symptoms to major complications such as sepsis and death related to the treatment (2).

Although TURBT plus intravesical BCG are the standard treatment for high grade NMIBC, intravesical chemotherapies are currently used after TURBT for adjuvant treatment of low grade NMIBC and also in some occasions for treatment of high grade NMIBC, in the case of BCG fail and finally as a recover therapy for patients that are ineligible for cystectomy. Mitomycin C (MMC), Doxorubicin, Valrubicin, Epirubicin, Thiotepa, Docetaxel, Gemcitabine, are the most used chemotherapy drugs for this goal (3).

Doxorubicin (DOXO), another component frequently used in Cisplatin-based regimens, is an antineoplastic drug of the anthracycline family that inhibits Topoisomerase II (4). DOXO is indicated for treatment of various cancers such as acute lymphoblastic leukemia, acute myeloid leukemia, transitional cell bladder carcinoma, breast carcinoma, neuroblastoma, Wilms tumor, ovarian carcinoma, thyroid carcinoma, prostate carcinoma, Hodgkin and non-Hodgkin lymphomas, sarcomas and Ewing sarcoma. DOXO has shown cardiotoxicity and can cause serious heart problems or life threatening. Moreover, it can cause a sharp decrease in the number of blood cells in bone marrow and an increase in the risk of leukemia (5).

Since the discovery of the biological activity of cisplatin [cis-diammine-dichloroplatinum (II)] the interest in anticancer drugs based on metals has for treatment of solid tumors has increased (6). Cisplatin forms a Platinum-DNA adducts at the N7 position of guanine, leading to intrachain crosslinkings 1, 2-d (GpG) and 1, 2-d (ApG) and interchain links, so activating proteins in response to injury. The proteins, in turn, inhibit cyclin-dependent kinase (CDK) and finally the cells undergo apoptosis via p53 (7).

Taking into account that bladder cancer (BC) is sensitive to both immunotherapies and chemotherapies, compounds that activate the immune system, including vaccines, biological response modifiers, tumor environment modulators of steroid hormones, can be considered potential candidates for the development of new treatments of BC to be used alone or in combination with systemic chemotherapies aiming to obtain greater therapeutic effect combined with lower toxicity. In animal models for study of cancer, P-MAPA (Protein aggregate magnesium-ammonium phospholinoleate-palmitoleate anhydride), a biological response modifier developed by Farmabrasilis a non-profit research network, obtained by fermentation from Aspergillus oryzae, has shown an ability to reverse the state of immunosuppression caused by tumoral processes, and such effect is linked to a significant therapeutic impact in the primary disease combined with low toxicity (8, 9).

Thus, the aims of this study were to evaluate and to compare P-MAPA intravesical immunotherapy in association with systemic chemotherapies (Doxorubicin and Cisplatin) for treatment of NMIBC in an appropriate animal model.

## MATERIALS AND METHODS

### Experimental Proceedings

Thirty-five female Fischer 344 strain rats, all 7 weeks old, with an average weight of 150grams, were provided by Multidisciplinary Center for Biological Investigation (CEMIB) at University of Campinas (UNICAMP). For induction of NMIBC, 30 animals were considered as Cancer group and anesthetized with 10% ketamine (60mg/kg, i.m.; Vibra® Roseira, São Paulo, Brazil) and 2% xylazine (5mg/kg, i.m.; Vibra® Roseira, São Paulo, Brazil), held in this state for 45 minutes to avoid spontaneous urination and administered 1.5mg/Kg dose of n-methyl-n-nitrosourea (MNU) dissolved in 0.30mL of sodium citrate (1M pH 6.0); every one via a 22-gauge angiocatheter intravesically every other week for 8 weeks (8, 9). The other 5 animals were considered as Control group. Two weeks after the last dose of MNU the occurrence of tumor was evaluated by cystography, as well as, considered clinical criteria such as haematuria and weight loss. Subsequently, the animals were divided into 7 groups (5 animals per group): Group 1 (Control): received 0.30mL dose of 0.9% physiological saline intravesically every other week for 6 weeks; Group 2 (MNU – Cancer): received the same treatment as the Group 1; Group 3 (MNU+P-MAPA): received 5mg/kg dose of P-MA-PA (Farmabrasilis, Campinas, SP, Brazil) intravesically every other week for 6 weeks (8, 9); Group 4 (MNU+Cisplatin): received 0.25mg/kg dose of cisplatin (Intas Pharmaceuticals, Ahmedabad, India) intraperitoneal, once per week for 4 consecutive weeks (10); Group 5 (MNU+Doxorubicin): received 3mg/kg dose of pegylated liposomal doxorubicin (Janssen, Johnson & Johnson, Horsham, PA, USA), intraperitoneal, every 15 days for 4 consecutive weeks (11); Group 6 (MNU+Cisplatin+P-MAPA): received concurrent treatment with P-MAPA and cisplatin at the same concentrations and the same routes of administration as Groups 3 and 4; Group 7 (MNU+Doxorubicin+P-MAPA): received concurrent treatment with P-MAPA and doxorubicin at the same concentrations and routes of administration as groups 3 and 5.

After 16 weeks of treatment, the animals were euthanized and their urinary bladder was collected and processed for histopathological and Western Blotting analyses.

The animal experiments described here were performed in accordance with the guidelines of the Brazilian College for Animal Experimentation (COBEA) and the guidelines set forth by our Institution (protocol number: CEUA/UNICAMP#3645-1).

### Histopathological Analysis

For histopathological analysis, fragments of urinary bladders were randomly collected from all animals in each group, fixed by immersion in Bouin, embedded in plastic polymer (Paraplast Plus; Sigma Chemical Co., St. Louis, MO, USA) cut into 5-μm thick and stained with hematoxylin-eosin. The neoplastic lesions were diagnosed using the nomenclature proposed by the World Health Organization/International Society of Urological Pathology consensus classification (12).

### Western Blotting Analysis: Akt (Protein kinase B), NF-kB (nuclear factor kappa-light-chainenhancer of activated B cells), PI3K (phosphatidylinositol-3-kinase), PTEN (Phosphatase and tensin homolog) and VEGF (Vascular endothelial growth factor)

Fragments of the urinary bladders were collected from 5 animals in each group, frozen in liquid nitrogen, weighed and homogenized in 50μL/mg of RIPA lysis buffer (EMD Millipore Corporation, Billerica, MA, USA). The tissue homogenized was centrifuged, and a sample of each extract was used for protein quantification by Bradford's method. Aliquots containing 70μg of protein were separated by SDS-PAGE on 10% or 12% polyacrylamide gels under reducing conditions. After electrophoresis, the proteins were transferred to nitrocellulose membranes and blocked with TBS-T containing 1% BSA (bovine serum albumin) and incubated at 4°C overnight with primary mouse monoclonal antibody sc-5298 (Santa Cruz Biotechnology, TX, USA) specific for Akt, rabbit polyclonal ab7970 (abcam, MA, USA) specific for NF-kB, rabbit polyclonal sc-67306 (Santa Cruz Biotechnology, TX, USA) specific for PI3K, rabbit monoclonal 138G6 (Cell Signaling, MA, USA) specific for PTEN and mouse monoclonal sc-53462 (Santa Cruz Biotechnology, TX, USA) specific for VEGF diluted in 1% BSA. The membranes were then incubated for 2h with anti-rabbit and anti-mouse secondary HRP-conjugated antibodies (diluted 1:3.000 in 1% BSA; Santa Cruz Biotechnology, TX, USA). Peroxidase's activity was detected by incubation with a diaminobenzidine chromogen. Western blots were run in duplicate, and urinary bladder samples were pooled from 5 animals per group for each repetition (10). The semi-quantitative densitometry (IOD - Integrated Optical Density) analysis of bands was conducted using NIH ImageJ 1.47v software (National Institute of Health, USA. Available in: http://rsb.info.nih.gov/ij/), followed by statistical analysis. β-actin (mouse monoclonal sc-47778, Santa Cruz Biotechnology, TX, USA) was used as endogenous positive controls for standardization of the readings of the band's staining intensity. The results were expressed as the mean±standard deviation of the ratio of each band's intensity to β-actin band intensity (9).

### Statistical analysis

For the statistical analysis, an analysis of variance (ANOVA) was used followed by Tukey's test for comparison of means. All analyses were performed with a significance level of P<0.01. The results were expressed as the mean±standard deviation.

## RESULTS

### Macroscopic Analysis: body weight

**Graph 1:**
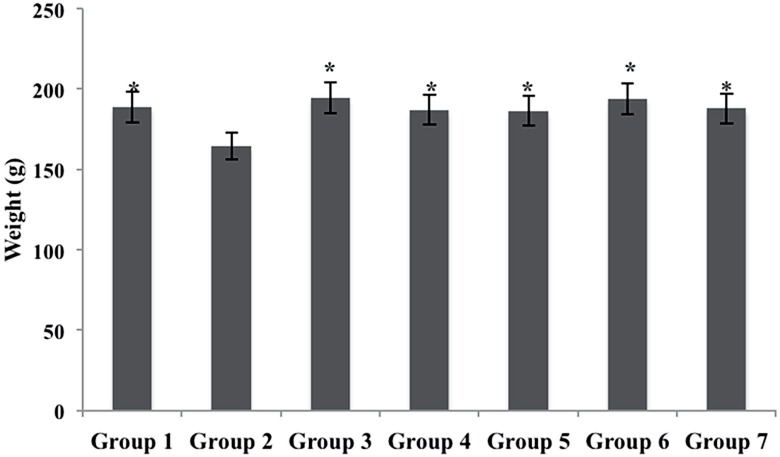
Body weight of the seven experimental groups.

The animals of the Group 2 (MNU - Cancer) showed a significant reduction in body weight when compared to other groups (Graph 1). In contrast, the animals of Groups 3, 4, 5, 6 and 7 showed no reduction in body weight when compared with Group 1 (Graph 1).

### Histopathological Analysis

**Figures 1a – 1g:**
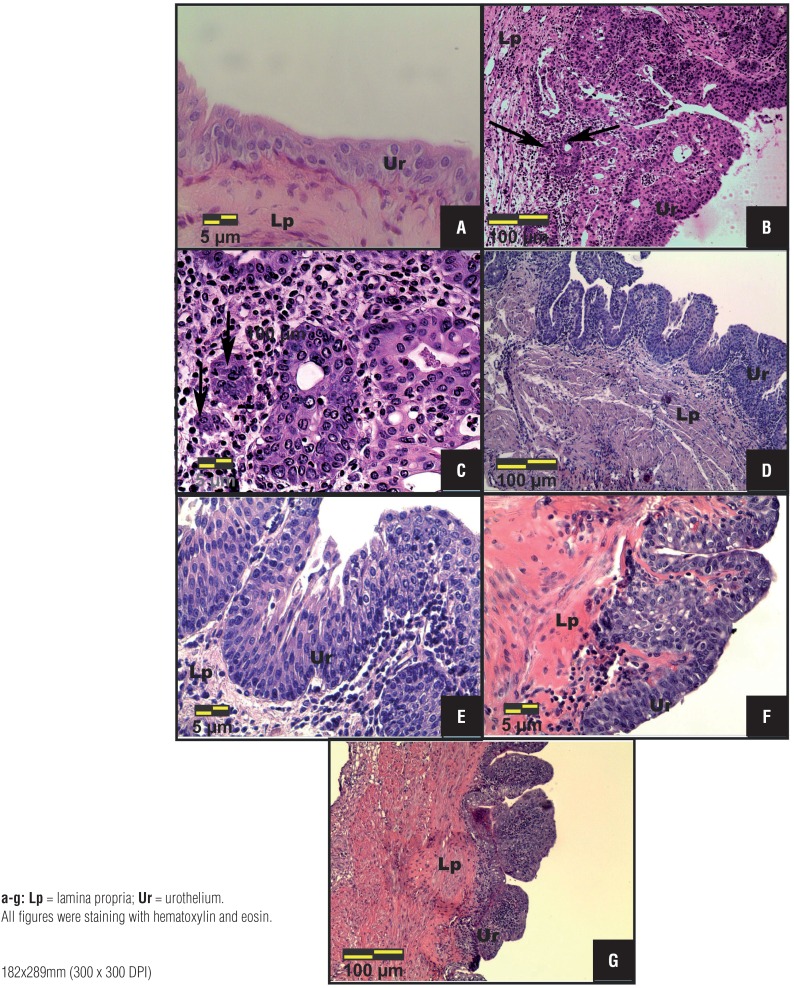
Photomicrographs of the most frequent histopathological changes in the urinary bladder of Control (a), MNU – Cancer (b, c), MNU+PMAPA (d, e) and MNU+Cisplatin (f, g) groups. (a) Normal urothelium, composed of 2-3 layers: a layer of basal cells, an intermediate layer of cells and a surface layer composed of apical or umbrella cells. (b, c) Tumor invading mucosa or submucosa of the bladder wall (pT1): neoplastic cells arranged in small groups (arrows) invading the lamina propria. (d, e) Papillary hyperplasia, characterized by thickening of the urothelium in the absence of cytologic atypia. (f) Flat carcinoma in situ (pTis) characterized by a disorderly proliferation of urothelial cells with cytologic atypia. (g) Papillary carcinoma in situ (pTa) characterized by extensive papillary lesions, urothelial cells with disordered arrangement and loss of polarity.

The animals of Group 1 did not show structural changes in their urinary tract, keeping the urothelium, formed by 2 – 3 layers, i.e., one basal cell layer, an intermediate cell layer, and a apical surface layer (Figure-1a). The Group 2 showed relevant structural changes such as hydronephrosis and hydroureter in the urinary tract; histopathological analysis of the urinary bladder of these animals showed tumor invading mucosa or submucosa of the bladder wall (pT1) in 20% and papillary carcinoma in situ (pTa) in 80% of animals (Figures 1b and 1c; Table-1).

**Table 1:** Percentage of histopathological changes in the urinary bladder of the seven experimental groups.

Urothelial lesions		Group 1 (n=5)	Group 2 (n=5)	Group 3 (n=5)	Group 4 (n=5)	Group 5 (n=5)	Group 6 (n=5)	Group 7 (n=5)
**No lesion**	Normal Urothelium	100% (5)	–	–	–	–	20% (1)	–
	Flat Hyperplasia	–	–	–	–	20% (1)	40% (2)	–
**Benign lesions**	Papillary Hyperplasia	–	–	80% (4)	–	–	20% (1)	–
	Flat Carcinoma *in situ* (pTis)	–	–	–	60% (3)	–	–	–
	Papillary Carcinoma *in situ* (pTa)	–	20% (1)	20% (1)	20% (1)	80% (4)	20% (1)	100% (5)
**Malign lesions**	Tumor invading mucosa or submucosa of the bladder wall (pT1)	–	80% (4)	–	20% (1)	–	–	–

**Group 1** (Control); **Group 2** (MNU-Cancer); **Group 3** (MNU+P-MAPA); **Group 4** (MNU+Cisplatin); **Group 5** (MNU+Doxorubicin); **Group 6** (MNU+Cisplatin+P-MAPA) and **Group 7** (MNU+Doxorubicin+P-MAPA).

The animals of Group 3 showed decrease of urothelial neoplastic lesions progression. Papillary hyperplasia (Figures 1d and 1e; Table-1), characterized by thickening of urothelium with no cytologic atypia was the most frequent lesion observed in 80% of animals; whereas pTa was observed in 20% of animals (Table-1).

After cisplatin treatment, the animals of Group 4 showed major structural changes in their urinary tract, such as hickening of the urinary bladder wall, increased urinary bladder vascularization and nodular lesions in the kidneys. The most frequent histopathological changes in the urinary bladder from this group were flat carcinoma in situ (pTis) (Figure 1f; Table-1), pTa (Figure 1g; Table-1) and pT1 in 60%, 20% and 20% of the animals, respectively (Table-1).

**Figures 2a – 2h:**
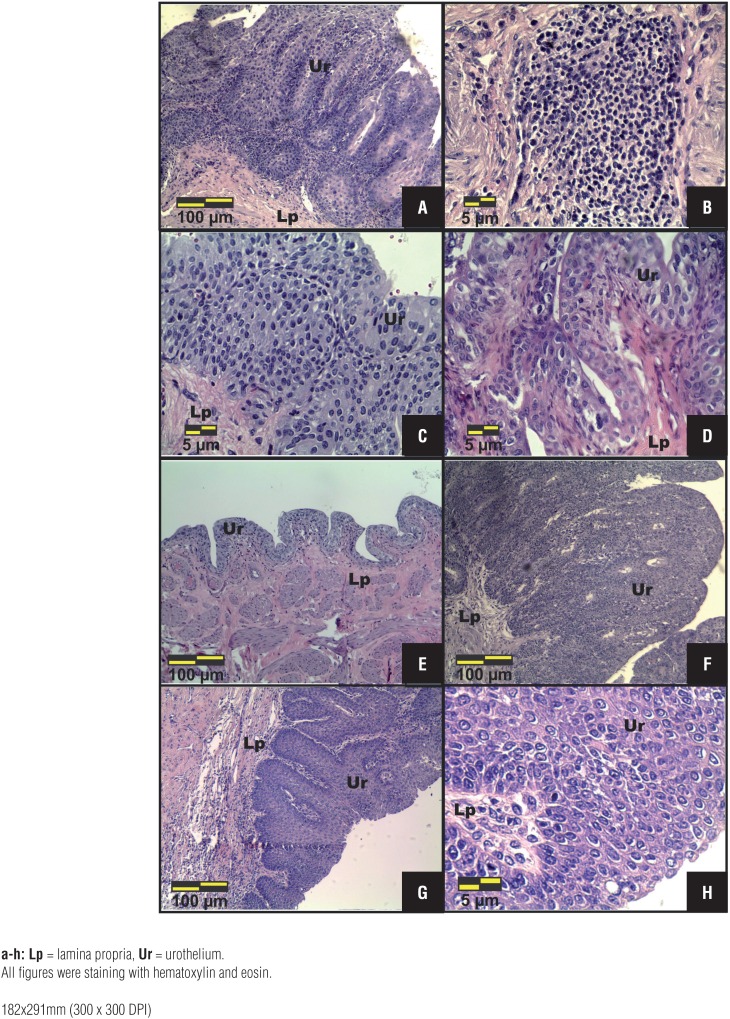
Photomicrographs of the most frequent histopathological changes in the urinary bladder of MNU+Doxorubicin (a, b), MNU+Cisplatin+P-MAPA (c, d, e) and MNU+ Doxorubicin+P-MAPA (f, g, h) groups. (a, f, g, h) Papillary carcinoma in situ (pTa). (b) pTa with inflammatory infiltrate in the lamina propria. (c) Flat hyperplasia characterized by thickening of the urothelium and absence of cytologic atypia. (d) Papillary hyperplasia. (e) Normal urothelium, similar to that shown in the Control group.

The animals treated with doxorubicin (Group 5) showed pTa (80%) with inflammatory infiltrate in the lamina propria (Figures 2a and 2b; Table-1) and 20% showed flat hyperplasia (Table-1). In addition, structural changes on the urinary tract as necrotic lesions in the kidneys and bilateral hydroureter were observerd in 80% of the animals.

Animals treated with P-MAPA associated to Cisplatin (Group 6) clearly showed better histopathological recovery from the cancer state than those observed on the Groups 3 and 4, showing decrease of urothelial neoplastic lesions progression in 80% of the animals (Figures 2c, 2d, 2e; Table-1). In contrast, the animals treated with cisplatin alone (Group 4) showed 100% of malignant lesions (Table-1). Normal urothelium was found in 20% of the animals from Group 6 (Figure-2e). The histopathological changes in this group were flat hyperplasia (Figure-2c), papillary hyperplasia (Figure-2d) and pTa in 40%, 20% and 20% of the animals, respectively (Table-1).

All animals from Group 7 (MNU+Doxorubicin+P-MAPA) showed 100% of (pTa) with squamous metaplasia associated (Figures 2f, 2g, 2h; Table-1).

### Western Blotting Analysis: Akt, NF-kB, PI3K, PTEN and VEGF

**Figures 3a – 3d:**
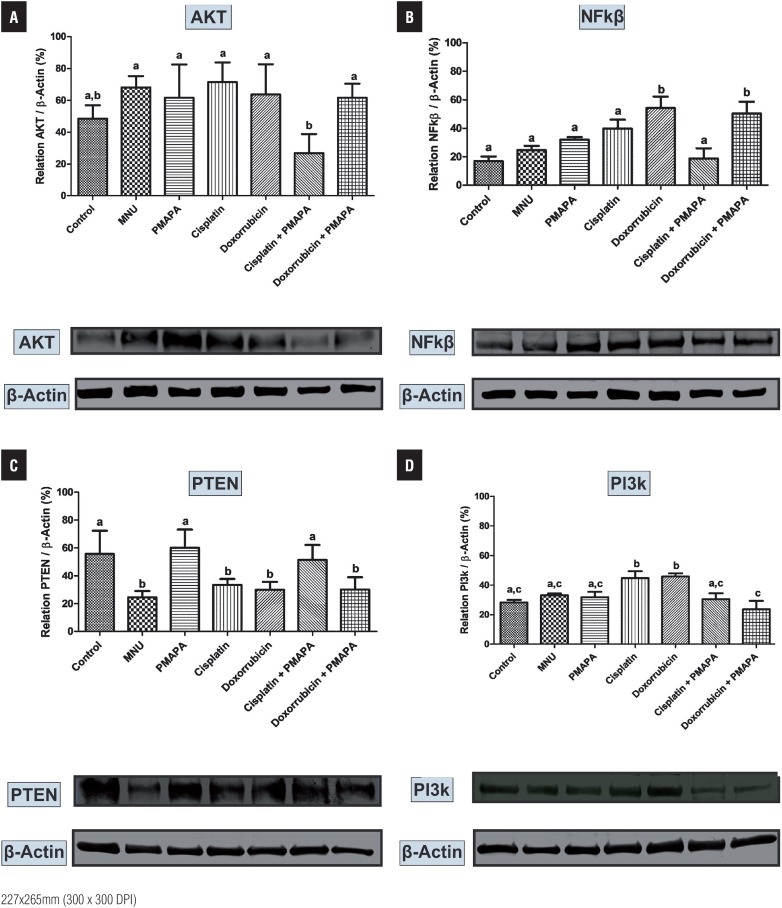
Western Blotting and semi quantitative determination of Akt (a), NF-kB (b), PTEN (c) and PI3K (d) protein levels. The protein levels were identified from the blots. β-Actin (42 kDa) was used as the internal control. Group 1 (Control), Group 2 (MNU – Cancer), Group 3 (MNU+PMAPA), Group 4 (MNU+Cisplatin), Group 5 (MNU+Doxorubicin), Group 6 (MNU+Cisplatin+PMAPA) and Group 7 (MNU+Doxorubicin+PMAPA). Data are expressed as the mean ± standard deviation (n= 5). Different lowercase letters (a, b, c, d) indicate significant differences (P < 0.01) between the groups after Tukey's test. Molecular weight: Akt (62 kDa), NF-kB (64 kDa), PTEN (54 kDa) and PI3K (190 kDa).

The lowest Akt protein levels were found in the Groups 1 and 6 (Figure-3a) when compared to other experimental groups, which were significantly higher for this protein (Figure-3a). NF-kB protein levels were significantly higher in the Groups 5 and 7 than in the other experimental groups (Figure-3b). Also, these levels were numerically lower in the Group 6 in relation to Groups 1, 2, 3 and 4 (Figure-3b).

The highest PTEN protein levels were found in the Groups 1, 3 and 6 in relation to other experimental groups, which were significantly lower for this protein (Figure-3c). In contrast, PI3K protein levels were significantly higher in the Groups 4 and 5 in relation to other experimental groups (Figure-3d).

**Figure 4:**
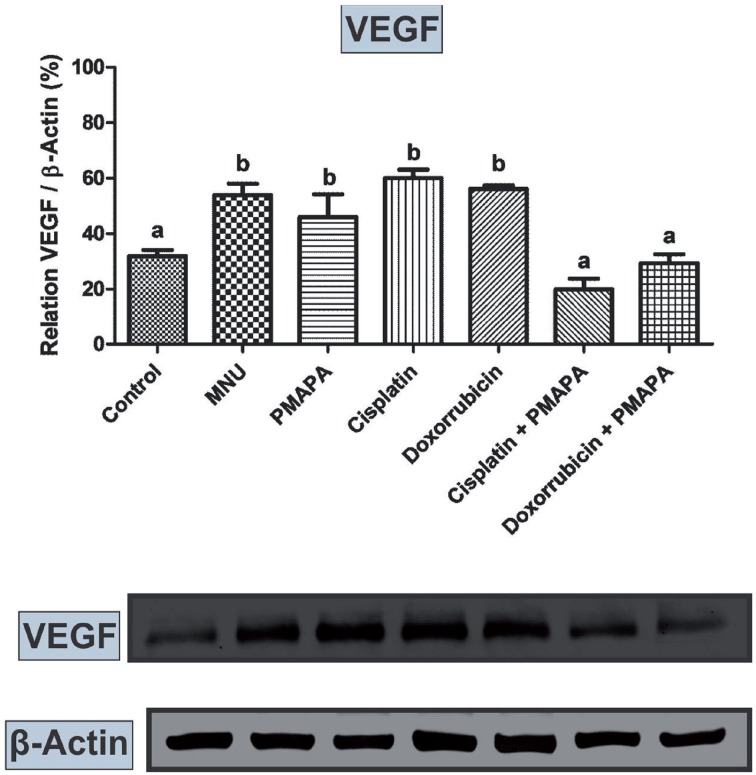
Western Blotting and semi quantitative determination of VEGF protein levels. The protein levels were identified from the blots. β-Actin (42 kDa) was used as the internal control. Group 1 (Control), Group 2 (MNU – Cancer), Group 3 (MNU+PMAPA), Group 4 (MNU+Cisplatin), Group 5 (MNU+Doxorubicin), Group 6 (MNU+Cisplatin+PMAPA) and Group 7 (MNU+Doxorubicin+PMAPA). Data are expressed as the mean ± standard deviation (n= 5). Different lowercase letters (a, b, c, d) indicate significant differences (P < 0.01) between the groups after Tukey's test. Molecular weight: VEGF (30 kDa).

VEGF protein levels were considerably lower in the Groups 1, 6 and 7 in relation to other experimental groups (Figure-4). In addition, these levels were numerically lower in the Group 3 in relation to Groups 2, 4 and 5 (Figure-4).

## DISCUSSION AND CONCLUSIONS

In this work, the histopathological results showed undifferentiated tumor, characterizing tumor invading mucosa or submucosa of the bladder wall (pT1) and papillary carcinoma in situ (pTa) in the MNU group, demonstrating that MNU was effective to induce NMIBC in this animal model. The P-MAPA intravesical immunotherapy alone led to a histopathological recovery and decrease of urothelial neoplastic lesions progression in 80% of the animals. The animals treated with systemic cisplatin or doxorubicin singly, showed drastic changes in the urinary tract, such as thickening of the urinary bladder wall, increased urinary bladder vascularization and nodular lesions on the kidneys, as well as, 100% of malignant lesions in the urinary bladder.

The groups that received P-MAPA intravesical immunotherapy combined to systemic therapies based on Cisplatin or Doxorubicin showed different results when compared to groups that received the same therapies administered singly. The histopathological changes were similar between intravesical P-MAPA plus systemic Cisplatin and intravesical P-MAPA alone treatments, showing decrease of urothelial neoplastic lesions progression and histopathological recovery in 80% of the animals. In contrast, systemic treatments with Cisplatin or Doxorubicin singly showed 100% of malignant lesions. Furthemore, the combined treatment with P-MAPA and Doxorubicin showed no decrease of urothelial neoplastic lesions progression and histopathological recovery.

BCG is the standard adjuvant treatment for high-grade NMIBC, and the only approved drug able to avoid or at least retard the progression to invasive disease (1). Despite therapeutic use of BCG for Tis and its adjuvant use for high-grade Ta or T1, disease recurrence occurs in up to 50% of patients (13). In addition, for Tis patients who are BCG non-responders the risk of progression is up to 90% at 6 months of treatment (13).

Following episodes of high grade NMIBC recurrence after BCG therapy, several conventional chemotherapy agents have been used including thiothepa, gemcitabine, mitomycin, gemcitabine plus mitomycin, docetaxel and valrubicin. In addition, immunotherapy (Interferonalpha or Interferon alpha-plus BCG) has also been used (13). Mycobacterium phlei cell wallnucleic acid complex (MCNA) has been proposed for intravesical treatment of NMIBC at high risk of recurrence or progression in patients who failed prior BCG immunotherapy (e.g., in patients who are BCG-refractory or BCG relapsing) and are not candidates for or refuse cystectomy (14). However, none of these drugs had been shown superiority over BCG and remains considered investigational (14). In the specific case of BCG-refractory CIS, Valrubicin, a semi-syntetic analog of doxorubicin, the only FDA-approved drug for treatment of such condition, shows effectivity in less of 10% of treated patients at 2 years and none with coincident stage T1 disease (15).

Therefore, the recommended standard of care for patients with recurrent high-grade disease after optimal BCG treatment has been to proceed with cystectomy (3, 16). However, patients who refuse or are unfit for bladder removal face an increased risk of progression to muscleinvasive disease (3, 16). No approved drug presenting similar or superior outcomes than BCG are available for treatment of this condition.

For treatment of invasive bladder cancer using a combination of TURBT, radiation therapy, and systemic chemotherapy-trimodality bladder preservation therapy-has been proposed and used in the last years for treatment of carefully selected patients (17). Also, the National Comprehensive Cancer Network Clinical Practice Guidelines in Oncology for Bladder Cancer and the International Consultation on Urological Diseases-European Association of Urology recognized the trimodality preservation bladder therapy as an alternative to radical cystectomy for patients with MIBC who are noncystectomy candidates and for those that are motivated to keep their native bladders (18). Despite the advances in the development of systemic chemotherapy regimens, the treatment of invasive and metastatic bladder cancer remains a big challenge, due the presence of toxicities associated with treatments as well the low rates of long-time survival for patients that present these conditions. Therefore, the need of development of new regimes, which provide better survival outcomes or similar survival with reduced toxicity, continues.

Although combinations of chemotherapies is the more frequent choice for the elaboration of treatment regimens for advanced and metastatic urothelial disease, the combination of chemotherapies with immunotherapies is also a possible approach to be used for the treatment of patients that are unfit for cystectomy as well for those presenting inoperable locally advanced and metastatic urothelial cancer and finally for the occasions that despite the presence of invasive disease an organ-conservative strategy may be used. For this reason while P-MAPA shows antitumor effects in animal model when used systemically in some types of cancer, such as Ehrlich ascitic tumor (19) and prostate cancer (20) in these studies our research group evaluate the drug candidate by intravesical via combined with systemic chemotherapy aiming to investigate the effects of such combination on NMIBC and also the possibility of its effect on the treatment of advanced or metastatic BC under a bladder preservation approach.

The reason for the selection of Cisplatin and DOXO for combined use with P-MAPA, by our research group is based on the knowledge that the main combinations developed to treat invasive or metastatic bladder cancer are cisplatin-based (21), such as the combination of methotrexate, vinblastine, doxorubicin, and cisplatin (MVAC). Developed some years ago (21) the combination MVAC remains until now the most used for the treatment of invasive and metastatic bladder cancer.

Cisplatin plus Gemcitabine is another cisplatin-based combination in advanced stage of development. Using such combination, a randomized phase III study enrolling patients with locally advanced or metastatic transitional cell carcinoma (TCC) of the urothelium indicates that the combination (GC) is an effective alternative to MVAC therapy presenting similar clinical efficacy with a better tolerability and safety profile (22).

Currently, DOXO can be administered intravenously at a 2mg/mL dose or intravesically at a 1mg/mL dose. The intravesical route of administration is used to treat monocytic carcinoma, papillary bladder tumors and carcinoma in situ to reduce recurrences after transurethral resection (5). However, administration of DOXO intravesical, though reducing adverse effects compared to intravenous administration, has low efficiency, and there is recurrence of tumor in 80% of cases. Taking in account that the mechanism of action of P-MAPA involves an interferon signaling pathway (9) in view of a study (23) showing a significant decrease of Interferon-gamma and IL-2 levels induced by Con A in splenocytes of doxorubicin treated mice, a hypothesis to be investigate, is that the systemic DOXO may depressed the IFN-gamma levels on treated animals, that may impaired the effects of P-MAPA.

In this work, Western Blotting analyses showed a decrease statistically significant in Akt, PI3K and NF-kB protein levels in MNU+Cisplatin+P-MAPA and MNU+P-MAPA groups in relation to other experimental groups. In contrast, PTEN protein levels were significant higher in MNU+Cisplatin+P-MAPA and MNU+P-MAPA groups than other experimental groups.

The PI3K/Akt/mTOR is a major intracellular signaling pathway responsible for promoting cell survival and proliferation (24). This pathway is initiated by PI3K enzyme, which is activated by tyrosine kinases or G-protein coupled receptors (24). PTEN protein acts as a phosphatase to dephosphorylate phosphatidylinositol (3–5) - trisphosphate (PtdIns (3–5) P3 or PIP3). PTEN specifically catalyses the dephosporylation of the 3 phosphate of the inositol ring in PIP3, resulting in the biphosphate product PIP2 (PtdIns (4, 5) P2) (24). This dephosphorylation is important because it results in inhibition of the PI3K and AKT signaling pathways, which can act as oncogenes for promoting cell survival (25). Akt activation leads to anti-apoptotic events and induces cell proliferation, which promotes carcinogenesis (26).

PTEN expression also induces apoptosis and suppresses cell growth through activation of caspase-3 and suppression of NF-kß (27). NFkB signaling pathway plays an important role in the responses to cancer, inflammation and stress (28). NF-kB is a transcription factor found in the cytoplasm bound to known NF-kB inhibitors (IκB). The activation of NF-kB by inflammatory and growth factors is mediated through the protein IκB kinases (IKKS), which phosphorylate IκB, resulting in ubiquitination and subsequent proteasomal degradation of IκBα (28). The wide distribution of NF-kB binding sites in the genome allows the regulation of a large number of genes and participates on fundamental cellular processes, such as apoptosis, proliferation, and differentiation (28).

Thus, it could be concluded that the association between P-MAPA intravesical immunotherapy and chemotherapeutic agents, especially cisplatin, promoted a reduction of PI3K/ Akt pathway activation and stimulated PTEN pathway, which led to reduced NF-kB levels with consequent decrease of proliferation of urothelial tumor cells.

Angiogenesis is a central process to tumor progression (29). VEGF is one of the key regulators of angiogenesis and the prime target of antiangiogenic drug development for the treatment of multiple cancers (29). Also, VEGF protects tumor cells against chemotherapy-induced apoptosis (29, 30). Therefore, the relative over expression of VEGF observed within the residual tumors after MVAC may reflect the clonal selection resulting from the death of tumor cells expressing low levels of VEGF and the survival of tumor cells expressing relatively high levels of VEGF, which rendered them relatively resistant to chemotherapy-induced apoptosis (30). In this work, we showed VEGF protein levels were significantly lower in the MNU+P-MAPA+Cisplatin and in the MNU+P-MAPA+Doxorubicin groups, as well as in the MNU+P-MAPA group, indicating that P-MAPA immunotherapy was essential in the inhibition of angiogenesis process, mainly when associated with systemic chemotherapeutic agents.

Although the recommended option in the case of BCG failures (e.g BCG refractory and BCG relapsing) is to proceed with early cystectomy, a conservative therapy must be tried for carefully selected patients, without unacceptable risks. In a translational perspective, taking into account previously published studies (8, 9) and the present data, intravesical P-MAPA immunotherapy may be considered as a valuable option for treatment of BCG unresponsive patients that unmet the criteria for early cystectomy.

For NMIBC patients that are unresponsive to BCG and simultaneously are unfit for or refuse cystectomy, the use of P-MAPA alone appears very promising because the data of this study shows that intravesical P-MAPA is clearly superior (80% responders) to intravesical anthracycline (DOXO) for treatment of high-grade NMIBC.

Finally, it could be concluded that combination of P-MAPA intravesical immunotherapy and systemic cisplatin in the NMIBC animal model was effective, well tolerated and showed no apparent signs of antagonism between the drugs.

Thus, these findings appears promising because permits consider also the investigational use of intravesical P-MAPA combined with systemic cisplatin or cisplatin-based combinations for treatment of NMIBC patients unresponsive to BCG that are unfit for cystectomy due the high risk of comorbidities, aiming to fight simultaneously intravesical (superficial) and invasive (metastatic) lesions, if any, at the time of treatment.

Despite the limitations of the animal model used, that not provides invasive lesions, the data of this work indicate that use of intravesical P-MAPA in combination with systemic cisplatin or cisplatin-based combinations may be thinking for development of combinations aiming the conservative treatment of invasive bladder cancer to be evaluated in other animal models or even as salvage therapy.
